# Impaired Mitochondrial Function in iPSC-Retinal Pigment Epithelium with the Complement Factor H Polymorphism for Age-Related Macular Degeneration

**DOI:** 10.3390/cells10040789

**Published:** 2021-04-02

**Authors:** Mara C. Ebeling, Zhaohui Geng, Rebecca J. Kapphahn, Heidi Roehrich, Sandra R. Montezuma, James R. Dutton, Deborah A. Ferrington

**Affiliations:** 1 Department of Ophthalmology and Visual Neurosciences, University of Minnesota, Minneapolis, MN 55455, USA; ebeli017@umn.edu (M.C.E.); kapph001@umn.edu (R.J.K.); smontezu@umn.edu (S.R.M.); 2 Stem Cell Institute, University of Minnesota, Minneapolis, MN 55455, USA; gengx027@umn.edu; 3 Department of Genetics, Cell Biology, and Development, University of Minnesota, Minneapolis, MN 55455, USA; 4 Histology Core for Vision Research, University of Minnesota, Minneapolis, MN 55455, USA; rohri002@umn.edu

**Keywords:** induced pluripotent stem cell, retinal pigment epithelium, age-related macular degeneration, complement factor H, mitochondrial function, inflammation

## Abstract

Age-related macular degeneration (AMD), the leading cause of vision loss in the elderly, is characterized by loss of the retinal pigment epithelium (RPE). While the disease mechanism remains unclear, prior studies have linked AMD with RPE mitochondrial defects and genetic polymorphisms in the complement pathway. This study used RPE generated from induced pluripotent stem cells (iPSC-RPE), which were derived from human donors with or without AMD and genotyped for the complement factor H (CFH) AMD high-risk allele (rs1061170, Y402H) to investigate whether donor disease state or genotype had a detrimental effect on mitochondrial function and inflammation. Results show that cells derived from donors with AMD display decreased mitochondrial function under conditions of stress and elevated expression of inflammatory markers compared to iPSC-RPE from individuals without AMD. A more pronounced reduction in mitochondrial function and increased inflammatory markers was observed in CFH high-risk cells, irrespective of disease state. These results provide evidence for a previously unrecognized link between CFH and mitochondrial function that could contribute to RPE loss in AMD patients harboring the CFH high-risk genotype.

## 1. Introduction

Age-related macular degeneration (AMD) is the leading cause of progressive and irreversible vision loss in the elderly, affecting approximately 30% of individuals over 75 years old [1]. Many risk factors have been linked to the clinical manifestations of AMD, including environmental insults, such as smoking and a high-fat diet [2], and genetic polymorphisms, which have been identified in 34 high-risk loci associated with AMD [3]. One of the most prevalent AMD-associated single-nucleotide polymorphism (SNP) occurs in the gene encoding complement factor H (CFH). The SNP in CFH (nucleotide position 1277, substitution of T to C, rs1061170), where a tyrosine is substituted for histidine at position 402 (Y402H) in the protein, is found in ~50% of AMD patients [4]. CFH is a negative regulator of the alternative complement pathway, which kills invading pathogens and promotes the clearance of debris and dead cells [5]. CFH protects host cells by downregulating the complement cascade, where inappropriate activation can cause chronic inflammation and lysis of host cells via the formation of a membrane attack complex (MAC) on cell outer membranes [6]. Biochemical analysis of the CFH Y402H mutant protein shows it has reduced ability to inhibit complement activation [7], a factor that could contribute to the 3- to 5-fold increased likelihood for developing AMD in individuals harboring the high-risk (CT or CC) allele compared with the low-risk (TT) genotype [8]. Along with CFH, SNPs in other genes of the complement system (CFI, C2, C3, C9, CFB) identified in AMD patients supports a role for chronic inflammation in promoting AMD pathology [9].

The visual decline experienced with AMD is caused by a functional loss in the retinal pigment epithelium (RPE) and subsequent death of photoreceptors. The RPE cell layer, located between retinal photoreceptors and the outer retinal blood supply of the choroid, is essential for the maintenance of retinal homeostasis. RPE cells fulfill many key functions, such as phagocytosis of shed photoreceptor outer segments, transport of nutrients from the choroid to the outer retina, and secretion of factors that are crucial for the structural integrity of the retina and choroid [10]. Previous reports of AMD-associated defects in RPE mitochondria, including decreased mitochondrial mass [11] and content of electron transport chain proteins [12], increased mitochondrial DNA (mtDNA) damage [13,14,15], and reduced mitochondrial function [16], provide strong evidence for RPE mitochondrial defects as a contributing factor in AMD pathology. Of relevance to this study is the report of higher mtDNA damage in the RPE tissue from AMD donors harboring the CFH high risk allele compared with the low risk genotype, indicating a potential link between the CFH high-risk polymorphism and mitochondrial health [17]. Since mitochondria are the main energy source for RPE [18], disruptions in mitochondrial function and subsequent lower energy production could cause RPE cell death, one of the hallmarks of AMD [19].

In this study, we generated RPE from induced pluripotent stem cells (iPSC) derived from multiple human donors characterized for the presence and severity of AMD and genotyped for the CFH risk SNP (rs1061170). This model system allowed us to investigate whether donor disease state or genotype had a detrimental effect on mitochondrial function and inflammation, two characteristics that have been linked to early AMD pathology [20]. iPSC-RPE derived from AMD donors have been shown to exhibit several key features associated with AMD [21,22,23]. Recent studies utilizing iPSC-RPE also have begun to define how the high-risk SNPs associated with AMD, including Age-Related Maculopathy Susceptibility 2/High-Temperature Requirement A1 (ARMS2/HTRA1) [24,25] and CFH [26,27], can change RPE function. The functional defects in iPSC-RPE revealed by these studies have not only provided new insight into disease mechanisms, but also raise the possibility of using iPSC-RPE as a drug development platform for the emerging field of pharmacogenomics.

Results from the current study provide novel mechanistic details about how donor disease state and genotype affect RPE function. When compared with iPSC-RPE derived from donors without AMD, cells made from AMD donors show decreased mitochondrial function and an elevated inflammatory marker. iPSC-RPE derived from donors harboring the CFH high-risk allele exhibited an even more pronounced reduction in mitochondrial function and increased markers of inflammation irrespective of disease state. This key result provides evidence for a mechanism that links CFH and mitochondrial function and has implications for understanding the connection between the CFH polymorphism and AMD, which was first identified in population genome-wide association studies. Furthermore, these data suggest that individuals harboring the CFH Y402H allele may benefit from treatments aimed at preserving or improving RPE mitochondrial function.

## 2. Materials and Methods

### 2.1. Human Tissue Procurement and Grading

De-identified donor eyes were obtained from the Minnesota Lions Gift of Sight (St. Paul, MN, USA,). Eyes are obtained with written consent of the donor or donor’s family for use in medical research in accordance with the Declaration of Helsinki. The Minnesota Lions Gift of Sight is licensed by the Eye Bank Association of America (accreditation #0015204) and accredited by the Food and Drug Administration (FDA) (FDA Identifier 3000718538). Donor tissue is considered pathological specimens and is therefore exempt from the process of Institutional Review Board approval.

Evaluation for the presence or absence of AMD was determined by a Board Certified Ophthalmologist (Sandra R. Montezuma) from stereoscopic fundus photographs of the RPE macula using the criteria (RPE pigment changes and the presence, size and location of drusen) established by the Minnesota Grading System (MGS) [28]. Disease stage and donor demographics, provided by the Minnesota Lions Gift of Sight, is included in Table 1.

### 2.2. Genotyping

Genomic DNA was extracted from graded donor retinal tissue using QIAmp DNA Micro kit (Qiagen; Hilden, Germany). DNA was quantified using Quant-iT PicoGreen dsDNA assay kit (ThermoFisher; Waltham, MA, USA). Samples were genotyped for the Complement Factor H (CFH) variant Y402H using allele-specific primers designed for the single nucleotide polymorphism (SNP) rs1061170. CFH-Y402H-F: TGAGGGTTTCTTCTTGAAAATCA, CFH-Y402H-R: CCATTGGTAAAACAAGGTGACA. PCR product purified with Gel PCR DNA fragments extraction kit (IBI Scientific; Shelton, CT, USA) was submitted for classic Sanger Sequencing (U of MN Genomics Core). Base calling was manually inspected using Sequence Scanner 2 software (Applied Biosystems; Foster City, CA, USA).

### 2.3. Culturing iPSC-RPE Cells

The derivation of induced pluripotent stem cell (iPSC) from human conjunctival cells and the differentiation of iPSC into RPE was performed as previously described [29]. iPSC-RPE cells from passage 3 were used for characterization and functional assays. Optimal cell number and timing for procedures was experimentally determined for each assay. Cell number and condition are indicated under each experimental protocol.

### 2.4. Immunofluorescence

iPSC-RPE cells (4 × 10^4^ cells/well) were grown on Matrigel-coated transwell filters (Costar®) for one month. Paraformaldehyde-fixed cells were blocked for one hour in 10% normal donkey serum and then incubated in primary antibody overnight. The reaction was visualized using appropriate secondary antibody. Cells were cover slipped with mounting medium containing 4’,6-diamidino-2-phenylindole (DAPI) (Vector Laboratories; Burlingame, CA, USA) and imaged with an inverted confocal microscope (Olympus FluoView FV1000).

### 2.5. Enzyme-Linked Immunosorbent Assay (ELISA)

iPSC-RPE cells (4 × 10^4^ cells/well) were seeded onto 6.5mm transwell inserts (Corning; Tewksburg, MA, USA) coated with Matrigel (Corning). After culturing for 5 weeks, samples were collected from apical and basal chambers 24hr after media change. ELISAs for Pigment Epithelium-Derived Factor (PEDF) (R & D Systems; Minneapolis, MN, USA), Vascular Endothelial Growth Factor-A (VEGF-A) (eBioscience; San Diego, CA, USA), Interleukin-6 (IL-6) (BD Biosciences; San Jose, CA, U.S.A), CFH (Abcam; Cambridge, United Kingdom) and C3a (BD Biosciences) were conducted according to the manufacturer’s protocols. Growth factor concentration was derived from a standard curve and normalized to chamber volume.

### 2.6. Western Blotting

Cell pellets were collected and lysed in RIPA buffer. Protein concentrations were determined with the Bicinchoninic acid assay using albumin as the standard. Proteins were resolved on SDS-PAGE gels (made with 2,2,2-Trichloroethanol). Before transferring proteins to low-fluorescence Polyvinylidene difluoride (PVDF) membranes using BioRad Turbo Blot, gels were activated with UV light. After transfer, membranes were imaged to allow for normalization to protein load. Membranes were incubated overnight with primary antibodies, Total OXPHOS Human WB cocktail (Abcam), Voltage-dependent Anion Channel (VDAC) (Cell Signaling; Danvers, MA, USA), or Cytochrome Oxidase IV (COX IV) (Cell Signaling). Secondary antibodies conjugated to horseradish peroxidase along with the Chemiluminescence kit (ThermoFisher) were used to visualize the immune reactions. Images were taken using a BioRad ChemiDoc XRS. Densitometry was performed using Image Lab software (BioRad, Hercules, CA, USA). Intensity of bands were normalized for protein load and to a standard run on each blot. Fold-change values were calculated from the average for No AMD or CFH low risk values.

### 2.7. RNA Extraction, cDNA Synthesis and Quantitative RT-PCR

RNA from iPSC-RPE cell cultures was extracted using RNeasy Mini kit (Qiagen). cDNA was synthesized from total RNA prepared from iPSC-RPE cultured cells as previously described [30]. Quantitative reverse transcription PCR was performed using BioRad iQ5 multicolor real time PCR detection system. Primer pairs used in the analyses are found in Table S1. The geometric mean of housekeeping genes, Hypoxanthine guanine phosphoribosyltransferase 1 (HPRT1) and Acidic ribosomal phosphoprotein P0 (ARBP), was used to calculate ΔCt for each gene of interest. To determine fold-change relative to No AMD (or CFH low-risk), ΔΔCt of each AMD iPSC-RPE (or CFH high-risk) line was calculated by subtracting the mean ΔCt of No AMD iPSC-RPE (or CFH low risk). A modified Livak method was used to calculate relative expression using efficiency for each primer.

### 2.8. Measurement of Metabolic Function Using XFe96 Extracellular Flux Analyzer

Analyses of metabolic function (Cell Energy Phenotype Test (CEPT), Cell Mito Stress Test (CMST), and Mito Fuel Flex Test (MFFT)) were performed on live cells using an XFe96 Extracellular Flux Analyzer (Agilent Technologies; Santa Clara, CA, USA). iPSC-RPE cells (4 × 10^4^ cells/well) were seeded in five wells of a 96 well plate and grown for 48hr prior to analysis. Assays were performed using protocols suggested by the manufacturer (Agilent Technologies) and as described in Figure S1. After performing the CEPT and MFFT assays, cells were stained with Hoescht 33342 and the cell count of each well was determined by imaging the cells using Cytation One Imaging Reader (BioTek; Winooski, VT, USA). Total cell count per well was used for normalization Oxygen Consumption Rate (OCR)/Extracellular Acidification Rate (ECAR) and OCR in the CEPT and MFFT assays, respectively. The total amount of protein (mg) per well was used for normalization of OCR in the CMST assay. In total, five technical replicates were used to determine the mean value for each donor.

The Bioenergetic Health Index (BHI) was calculated from the data produced with the CMST assay using the formula: BHI = log ((Spare Respiratory Capacity * ATP-linked production)/(Non-Mito Oxygen Consumption * Proton Leak)) [31].

### 2.9. Statistical Analysis

Data were prescreened for outliers using a Grubb’s test and the single largest outlier was removed when indicated by the test results. All data sets were tested for normal distribution. If data were normally distributed, a Student’s t-test was performed. If the dataset did not pass the normality test, a Mann–Whitney non-parametric test was used. Statistical parameters, including types of tests, number of samples (*n*), and significance are reported in the figures and figure legends. Statistical analyses were performed using GraphPad Prism 8. Data were presented as mean ± SEM. *p* < 0.05 was considered statistically different and *p* < 0.1 was considered a trend.

## 3. Results

### 3.1. Donor Demographics for iPSC-RPE

The somatic cell source for the iPSC-RPE used in this study were epithelial cells from the conjunctiva of adult human donor eyes. Clinical information, demographics, and CFH genotype (rs1061170, Y402H risk SNP) for all donors are provided in Table 1. The presence and severity of AMD was determined from high-resolution photomicrographs of donor retina using the Minnesota Grading System (MGS) [28]. Donors included those with No AMD (MGS1) and donors with early (MGS2) or intermediate (MGS3) disease stage, combined to form the AMD group. Our rationale for combining these two groups is based on their equivalent response for mitochondrial function (Figure S2). The average age of donors with No AMD (70 ± 9.5 year) and AMD (75 ± 8.1 year) was not statistically different (*p* = 0.13). Reprogramming primary conjunctival cell cultures and subsequent differentiation into RPE generated 14 iPSC-RPE lines from 10 No AMD donors (No AMD iPSC-RPE) and 24 lines from 16 AMD donors (AMD iPSC-RPE). Classification of donors based on their CFH genotype provided a comparison of donors harboring the low-risk (TT) versus high-risk (CC and CT) allele.

### 3.2. Characterization of iPSC-RPE

**Figure 1:**
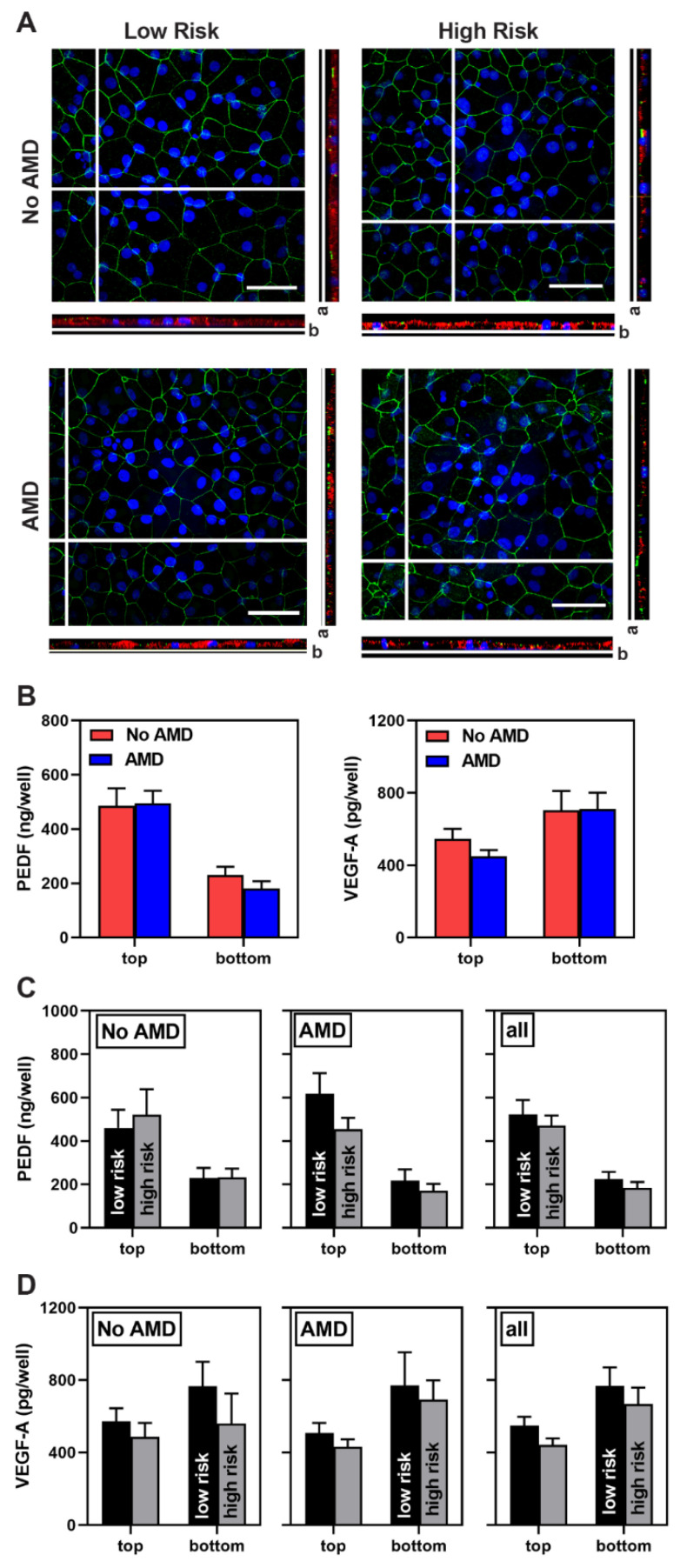
Characterization of iPSC-RPE derived from epithelial cells of the conjunctiva. (**A**) En Face views of the immunostained iPSC-RPE monolayer showing ZO-1 (green) marking cell borders and the orthogonal x-z views showing Bestrophin (red). Direction of the apical (a) and basal (b) side of the orthogonal image are indicated. Scale bar = 40 um. Nuclei are stained using DAPI (blue). (**B**–**D**) Results from ELISA analysis of PEDF and VEGF-A content measured in apical (top) and basal (bottom) media from iPSC-RPE grown in transwells. Data show results comparing (**B**) No AMD (7 donors, 10 lines) and AMD (12 donors, 17 lines) donors, and (**C**,**D**) donors genotyped for the CFH low-risk (No AMD 4 donors, 6 lines; AMD 3 donors, 4 lines) and CFH high-risk (No AMD 3 donors, 4 lines; AMD 9 donors, 13 lines) alleles. PEDF = pigment epithelium-derived factor, VEGF-A = vascular endothelial growth factor A. Data are presented as mean ± SEM. Unpaired t-tests was used to compare data from No AMD to AMD (B) or high- to low-risk (**C**,**D**). All comparisons were not statistically different.

As we have shown previously, iPSC-RPE exhibit characteristics of native RPE [29]. Confluent iPSC-RPE cell lines were pigmented and displayed a cobblestone appearance (Figure S3A). Immunofluorescent images show cells attain proper polarization and form tight junctions, as confirmed by the basal localization of Bestrophin (BEST1), an RPE-specific calcium-activated chloride channel [32], and staining at cell margins for zonula occludens (ZO-1) (Figure 1A). Assessment of gene expression for the RPE-specific proteins BEST1 and RPE65 showed no difference between cells from No AMD and AMD donors (Figure S3B). iPSC-RPE grown on transwells secreted pigment epithelium-derived factor (PEDF) preferentially to the apical side of the monolayer and vascular endothelial growth factor-A (VEGF-A) preferentially to the basolateral side (Figure 1B–D). Content of these growth factors, as well as overall cell morphology, revealed no disease- or genotype-dependent differences.

### 3.3. Metabolic Dysfunction in AMD and High-Risk iPSC-RPE

**Figure 2:**
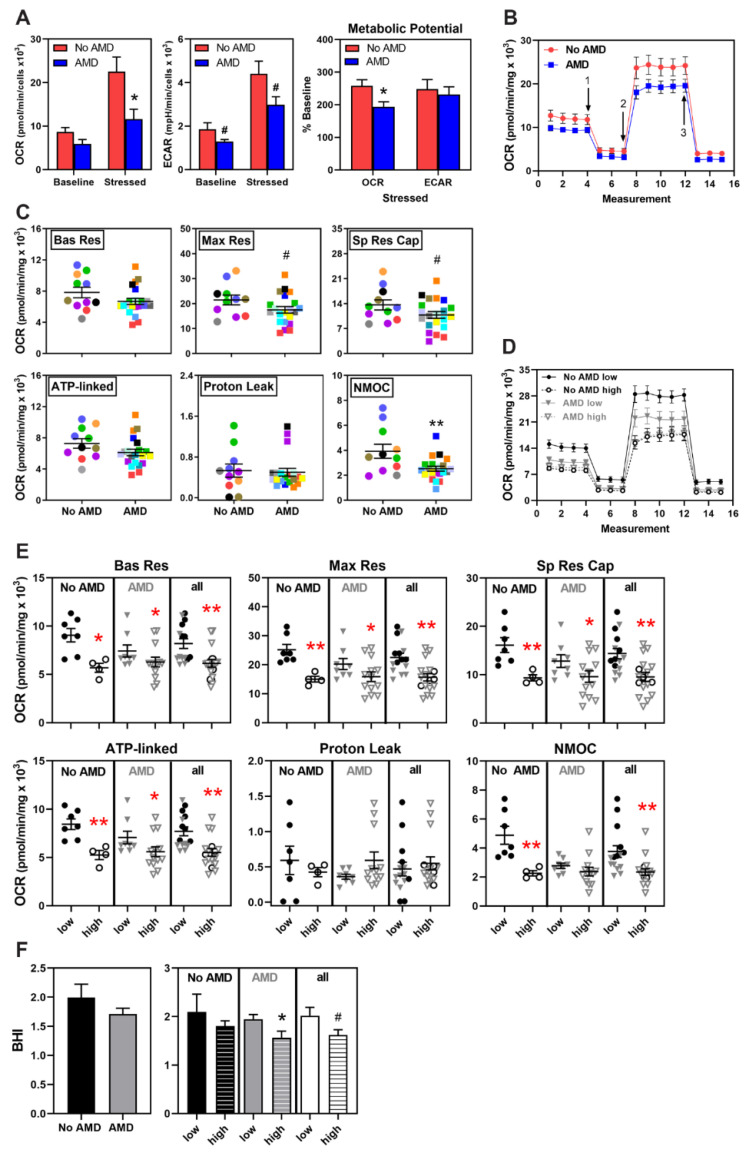
Metabolic dysfunction in AMD and high-risk iPSC-RPE. (**A**) Results from Cell Energy Phenotype Test of iPSC-RPE from No AMD (*n* = 4) and AMD (*n* = 6) donors shows the baseline and stressed OCR and ECAR. Metabolic Potential (in percent) was calculated from the change in OCR and ECAR. (**B**) Trace from the Cell Mito Stress Test (CMST) of OCR for iPSC-RPE derived from No AMD (8 donors, 11 lines) and AMD (15 donors, 21 lines) donors. Arrows indicate injection of oligomycin (1), FCCP (2), and antimycin A plus rotenone (3). (**C**) Parameters of mitochondrial function were calculated from data shown in B. Lines from the same donor are shown by the matched color symbols. (**D**) Trace from CMST assay showing OCR for iPSC-RPE from CFH low-risk (closed symbols, No AMD 5 donors, 7 lines; AMD 5 donors, 8 lines) and high-risk (open symbols, No AMD 3 donors, 4 lines; AMD 7 donors, 13 lines) donors. (**E**) Parameters of mitochondrial function calculated from data shown in D. Black symbols = No AMD, Grey symbols =AMD. Bas Res = Basal Respiration; Max Res = Maximal Respiration; Sp Res Cap = Spare Respiratory Capacity; ATP-linked = ATP-linked respiration; NMOC = Non-Mitochondrial Oxygen Consumption. (**F**) Bioenergetic Health Index (BHI) was calculated using the data from C and E. Unpaired t-tests were used to determine statistical significance in (**A**,**C**,**E**,**F**). Data are mean ± SEM. * *p* < 0.05, ** *p* < 0.01, # *p* = 0.1 is considered a trend. All other comparisons were not statistically different. See Figure S1 for a graphical description of assays in A and B.

We have previously reported primary RPE cultures from AMD donors exhibit lower mitochondrial function and glycolytic capacity [16]. In this study with iPSC-RPE, to compare metabolic function in cells derived from No AMD and AMD donors, we performed a Cell Energy Phenotype Test (CEPT) using an XFe96 Extracellular Flux Analyzer. (See Figure S1A for description). This assay simultaneously measures two major energy producing pathways—Mitochondrial respiration and glycolysis. At baseline, iPSC-RPE from both No AMD and AMD donors had quiescent phenotypes (low Oxygen Consumption Rate (OCR) and low Extracellular Acidification Rate (ECAR)) (Figure S1A). Once metabolically stressed with oligomycin and Carbonyl cyanide-p-trifluoromethoxyphenylhydrazone (FCCP), both groups increased OCR and ECAR in response to the change in energy demand (Figure 2A and Figure S1A). However, there was a significant difference in the degree of activation. In No AMD iPSC-RPE, OCR and ECAR increased more than two-fold under stress, while AMD iPSC-RPE had a more modest response, as reflected in lower OCR (*p* = 0.02), ECAR (*p* = 0.07), and metabolic potential (*p* = 0.03) (Figure 2A).

To obtain a more comprehensive assessment of the iPSC-RPE bioenergetic profile, we measured mitochondrial function and fuel oxidation using the Cell Mito Stress Test (CMST) and the Mito Fuel Flex Test (MFFT), respectively. (See Figure S1B,C for description). Figure 2B shows a trace of the average OCR for iPSC-RPE from No AMD and AMD donors, which was used to calculate the mitochondrial functional parameters for individual cell lines (Figure 2C). In general, AMD iPSC-RPE had lower basal respiration (−15%), max respiration (−19%), spare respiratory capacity (−21%), ATP-linked respiration (−16%), and non-mitochondrial respiration (−36%) compared with No AMD iPSC-RPE (Figure 2C). However, due to variability within each group, only the reduction in non-mitochondrial respiration reached statistical significance (*p* = 0.007) with a trend observed for max respiration and spare respiratory capacity (*p* = 0.09). Of note, measurements from duplicate cell lines derived from individual donors showed concordance; for example, variance in basal respiration and ATP-linked respiration averaged ~8 ± 2.0% (mean ± SEM) and maximal respiration was 12 ± 2.6% between cell lines derived from the same somatic cell donor (Figure S4).

To evaluate whether CFH genotype affects mitochondrial function, we sorted the data based on the presence (CT and CC) or absence (TT) of the CFH high-risk allele for each disease state and then by combining all the data to show the effect of genotype, irrespective of disease. Figure 2D shows a trace of the average OCR measurements in iPSC-RPE from CFH low- and high-risk donors. Irrespective of disease state, cells with the CFH high-risk genotype had significantly lower basal respiration, maximal respiration, spare respiratory capacity, and ATP-linked respiration than cells with the low-risk genotype (Figure 2E).

The Bioenergetic Health Index (BHI) provides an overall assessment of mitochondrial health by distilling multiple metrics from the CMST into a single value [31]. Higher BHI indicates healthier mitochondria. The average BHI of AMD iPSC-RPE (BHI = 1.7) trended 15% lower than for No AMD iPSC-RPE (BHI = 2), which is consistent with decreased mitochondrial health with AMD (Figure 2F). Considering the CFH genotype, AMD iPSC-RPE from donors with the high-risk allele had a significantly lower (20%) BHI compared with AMD iPSC-RPE from donors with the low-risk allele (*p* = 0.03; Figure 2F). This difference was also observed when comparing all high-risk to low-risk donors; BHI was 20% lower in the high-risk group (*p* = 0.06). These findings, combined with the results of the CMST assay, indicates that mitochondrial function is compromised in iPSC-RPE derived from donors with CFH high-risk allele regardless of AMD disease status.

**Figure 3:**
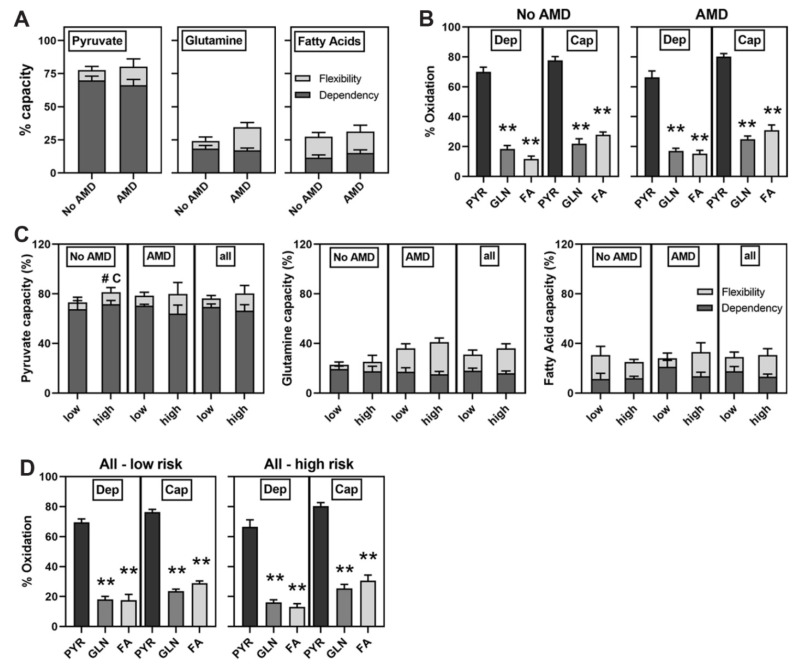
No difference in substrate utilization. Mito Fuel Flex Test results for iPSC-RPE (**A**,**B**) from No AMD (6 donors, 7 lines) and AMD (11 donors, 14 lines) donors and (**C**,**D**) from CFH low-risk (No AMD 3 donors, 3 lines, AMD 4 donors, 5 lines) and CFH high-risk (No AMD 3 donors, 4 lines; AMD 7 donors, 9 lines). (**B**,**D**) Graphs show metabolic substrate dependency (Dep) and capacity (Cap) for PYR = pyruvate, GLN = glutamine, FA = Fatty Acids. Unpaired *t*-tests were used to determine if capacity and dependency were different (**A**) between No AMD and AMD or (**C**) between low-risk and high-risk. One-Way ANOVA with Tukey’s post-hoc test was used to determine if there was a difference in fuel preference (**B**,**D**). All data are mean ± SEM. ** *p* < 0.01 in (**B**,**D**), # *p* < 0.1 for capacity (#C) in (**C**). All other comparisons were not statistically different. See Figure S1 for a graphical description of assay.

Mito Fuel Flex Tests were performed to investigate mitochondrial fuel utilization of long-chain fatty acids, pyruvate, and glutamine. This test revealed no disease-dependent difference in capacity, defined as the ability to use a specific fuel, or dependency, which is the requirement for either pyruvate, glutamine or fatty acids (Figure 3A). However, it did reveal that iPSC-RPE from both groups prefer pyruvate (*p* < 0.01) as shown by the significantly higher oxidation of pyruvate compared with glutamine and fatty acids (Figure 3B). We also investigated if differences in mitochondrial fuel utilization could help explain the lower mitochondrial function observed in cells harboring the CFH high-risk allele (Figure 2). However, data show the CFH genotype had no impact on fuel utilization except for a slightly increased capacity (12%) for pyruvate oxidation in the No AMD high-risk compared to low-risk groups (*p* = 0.09) (Figure 3C,D). Overall, our results suggest differences in fuel utilization did not contribute to the decreased mitochondrial respiration in iPSC-RPE generated from CFH high-risk donors.

### 3.4. No Significant Difference in Mitochondrial Proteins

**Figure 4:**
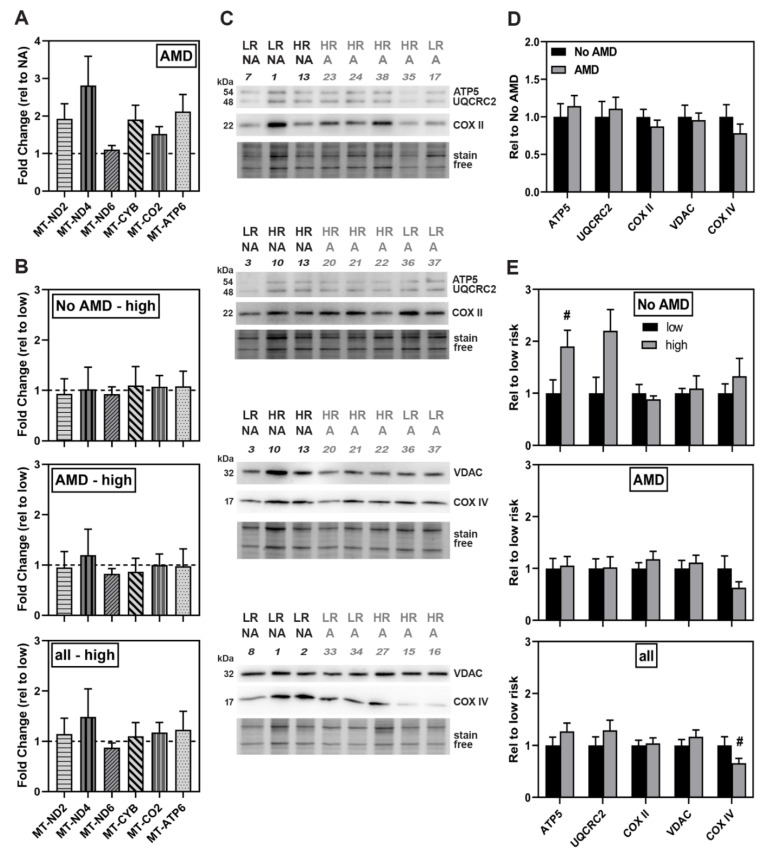
No significant difference in gene expression or content of mitochondrial-related proteins. (**A**,**B**) Gene expression analysis by qRT-PCR of OXPHOS genes in (A) iPSC-RPE from No AMD (5 donors, 8 lines) and AMD donors (13 donors, 20 lines) or (**B**) CFH low-risk (No AMD 3 donors, 5 lines; AMD 5 donors, 8 lines) and high-risk (No AMD 1 donor, 2 lines; AMD 8 donors, 12 lines). Results are fold-change in expression relative to the average for (**A**) No AMD donors (dashed line) or (**B**) high-risk relative to the average of low-risk samples (dashed line). In (**A**) and (**B**), there were no significant differences as assessed by un-paired t-tests of ΔΔCt values. (**C**) Representative image of western blots used to analyze protein content of iPSC-RPE cells. NA = No AMD, A = AMD. LR = low risk, HR = high risk. Numbers correspond with specific donor lines listed in Table 1. Stain free image below blot was used to normalize protein load. (**D**,**E**) Content analysis of mitochondrial-related proteins in (D) No AMD (7 donors, 9 lines) and AMD (13 donors, 21 lines) iPSC-RPE or (**E**) Low-risk (No AMD 4 donors, 5 lines; AMD 5 donors, 8 lines) and high-risk (No AMD 3 donors, 4 lines; AMD 8 donors, 13 lines). Data were normalized to the mean density for (**D**) No AMD iPSC-RPE or (**E**) low-risk iPSC-RPE. All data are mean ± SEM. # *p* < 0.1 determined by un-paired t-test. All other comparisons were not statistically different.

Factors that could influence mitochondrial function include differences in the content of proteins involved in oxidative phosphorylation (OXPHOS) and the total amount of mitochondria present in the cell. Quantitative assessment of expression for genes of respiratory complexes I, III, IV, and V showed no significant disease-dependent difference (Figure 4A). Likewise, no differences in the levels of OXPHOS gene expression were observed when comparing low- versus high-risk genotypes (Figure 4B).

Western blot analysis was performed to evaluate content of three OXPHOS proteins, ATP synthase subunit 5 (ATP5), Cytochrome b-c1 complex subunit 2 (UQCRC2), and cytochrome C oxidase subunit 2 (COX II) and two other mitochondrial membrane proteins (Figure 4C). Quantitative assessment of the immune reactions for OXPHOS proteins showed similar content in both No AMD and AMD iPSC-RPE (Figure 4D). As an estimate of mitochondrial number, we measured the content of voltage-dependent anion channel (VDAC) and cytochrome c oxidase subunit IV (COX IV), proteins located on the outer and inner mitochondrial membranes, respectively. We found content was similar between No AMD and AMD iPSC-RPE (Figure 4D).

Evaluating the protein data based on genotype (Figure 4E) showed similar content of OXPHOS proteins across groups except for ATP5, which was 1.5-fold higher in the No AMD high-risk group (*p* = 0.07). When comparing the content of VDAC and COX IV, no differences based on genotype for No AMD and AMD were observed. For the combined high- and low-risk groups, we found that only the content of COX IV was slightly lower in high-risk compared to low-risk iPSC-RPE (*p* = 0.09). Taken together, these data suggest mitochondrial protein expression and content are not significantly influenced by either disease or genotype.

### 3.5. Altered Expression of Complement Pathway and Markers of Inflammation in AMD and High-Risk iPSC-RPE

The presence of the CFH risk allele can lead to prolonged complement activation and subsequent chronic inflammation [6]. Transcriptional expression of the alternative complement pathway components (C3; complement component 3a receptor, C3AR; complement component fragment C5a receptor, C5AR1; pathway regulators, Complement Factor B, CFB; CFH, CFI, CD46, CD55, and the MAC complex inhibitor CD59) and markers of inflammation (Monocyte Chemoattractant Protein-1 (MCP-1), Tumor Necrosis Factor alpha (TNFα), IL-1β, IL-6) were measured under basal conditions to determine the effect of disease state and CFH genotype.

**Figure 5:**
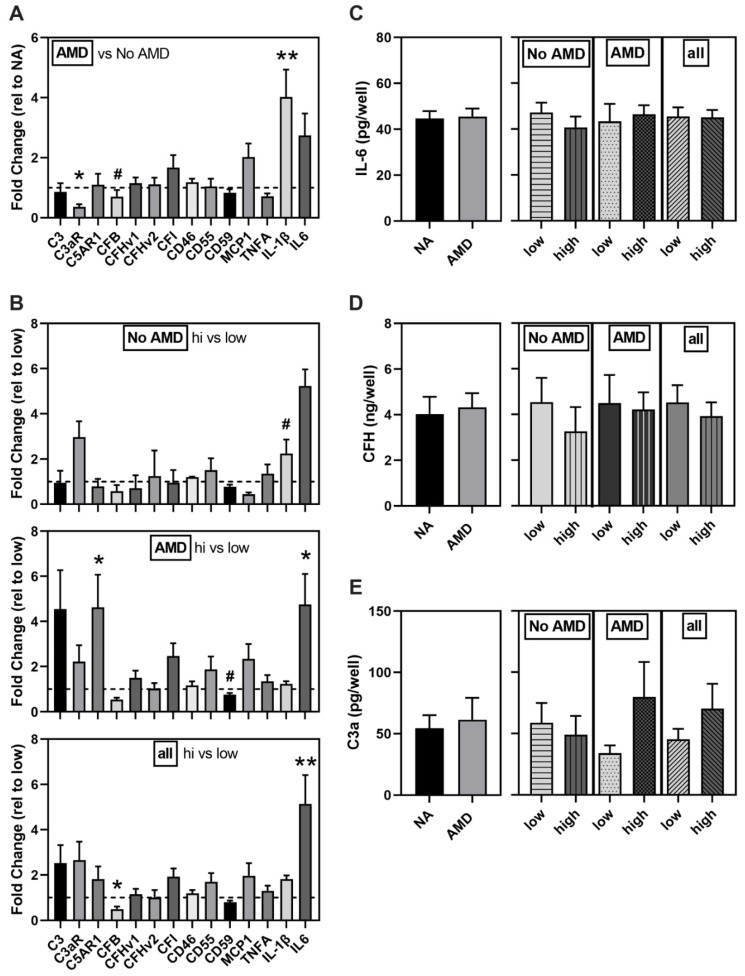
Altered markers of inflammation and complement components in AMD and high-risk iPSC-RPE. (**A**,**B**) Gene expression analysis of complement-related genes in iPSC-RPE from (**A**) No AMD (5 donors, 8 lines) and AMD (13 donors, 20 lines) donors or (**B**) CFH low-risk (No AMD *n* = 3 donors, 5 lines; AMD *n* = 5 donors, 8 lines) and high-risk (No AMD *n* = 1 donor, 2 lines; AMD *n* = 8 donors, 12 lines). Results shown are fold-change in expression of (**A**) AMD relative to the average of No AMD samples (dashed line) or (**B**) high-risk relative to the average of low-risk donors (dashed line). ELISA for IL-6 (**C**), CFH (**D**), and C3a (**E**) in media from iPSC-RPE comparing No AMD (7 donors, 10 lines) and AMD (12 donors, 17 lines) donors or low risk (No AMD 4 donors, 6 lines; AMD 4 donors, 5 lines) and high risk (No AMD 3 donors, 4 lines; AMD 9 donors, 13 lines) donors. NA = No AMD. All data are mean ± SEM. ** *p* < 0.01, * *p* < 0.05, # *p* < 0.1 determined by un-paired t-tests. All other comparisons were not statistically different.

Comparing expression between disease states, AMD iPSC-RPE showed lower expression of C3aR (0.6-fold, *p* = 0.01) and CFB (0.3-fold, *p* = 0.052) and 4-fold higher expression of the cytokine, IL-1β (*p* = 0.001) (Figure 5A).

Evaluating gene expression data based on genotype showed high-risk donors without AMD had higher expression of the cytokine IL-1ꞵ (2-fold, *p* = 0.09). High-risk iPSC-RPE from AMD donors had decreased expression of CD59 (0.8 fold, *p* = 0.09) and increased expression of C5AR1 (4.6 fold, *p* = 0.03) and IL-6 (4.8 fold, *p* = 0.05) compared to the low-risk group (Figure 5B). When combining all iPSC-RPE lines regardless of AMD disease status, we found CFB expression was significantly lower (0.5 fold, *p* = 0.04) and IL-6 expression was significantly higher (5-fold, *p* = 0.006) in iPSC-RPE derived from donors with the high-risk genotype. Note that while mean expression of several genes had a greater than two-fold change, the high within-group variability prevented reaching statistical significance.

ELISA was used to determine if the observed increase in IL-6 gene expression translated to increased secreted IL-6 protein content. We observed IL-6 content were similar when comparing disease state and genotype (Figure 5C). We also measured the secreted levels of CFH (Figure 5D) and C3a (Figure 5E) and found content was also similar between groups irrespective of disease state or genotype.

## 4. Discussion

In this study, we used iPSC-RPE derived from multiple human donors graded for the presence or absence of AMD and also genotyped for the CFH Y402H allele associated with a high risk of developing AMD. This model system allowed us to perform an extensive comparison of mitochondrial function between RPE lines to determine disease- and genotype-dependent changes. Our results show that under basal conditions, RPE derived from donors with AMD displayed a ~20% reduction in mitochondrial function (Figure 2C), which while consistent with our previous report in primary RPE cultures [16], did not reach statistical significance. In contrast, application of stress induced by mitochondrial inhibitors revealed a significant reduction in the ability of AMD iPSC-RPE to respond to increased energy demands (Figure 2A). Examining data based on the CFH genotype revealed even more dramatic differences in mitochondrial function. iPSC-RPE from donors harboring the high-risk allele showed significant reductions in all mitochondrial functional parameters (Figure 2E), as well as in overall mitochondrial health (Figure 2F), irrespective of the donor’s disease state. Since there were no differences in energy utilization (Figure 3) or reduction in mitochondrial content (Figure 4), these findings highlight a potential novel role for the complement pathway in regulating mitochondria that may be mechanistically linked to AMD disease susceptibility and progression.

We also evaluated how disease state and genotype affected expression of complement pathway genes and markers of inflammation. The observed minor changes in complement genes associated with disease and risk genotype (Figure 5A,B) and similar levels of secreted CFH and C3a (Figure 5D,E) did not provide a consistent picture of either over or under activation of this pathway. A total of four inflammatory markers were interrogated to gauge if the cells were exhibiting an inflammatory response under basal conditions. Our results show elevated gene expression of IL-1β and IL-6 is associated with AMD and the CFH high-risk genotype, respectively (Figure 5A,B). The expression of these acute-phase inflammatory cytokines is regulated by the synergistic action of a number of transcription factors, including Nuclear Factor-κ-B (NFκB), AP1, Cre-binding protein, Nuclear Factor-IL-6, and Sp-1 [33,34]. Activation of these transcription factors is induced by multiple stimuli, such as the inflammatory molecules: lipopolysaccharide (LPS) and TNFα, or oxidative stress. Thus, the elevated expression of IL-1β and IL-6 may reflex the presence of one or more conditions that activate relevant transcription factors. Of note, elevated IL-6 gene expression did not translate into higher IL-6 protein as there was no difference in the amount of secreted IL-6 protein between groups (Figure 5C). IL-6 is a constitutively secreted protein that undergoes co-translational import into the endoplasmic reticulum, is trafficked to the Golgi complex, and then loaded into endosomes for transport to the plasma membrane [35]. In activated immune cells, the apparent bottleneck for cytokine secretion occurs during post-Golgi trafficking, which was the rate limiting step for cytokine release after pathogenic stimuli [35]. While this mechanism has not been thoroughly investigated in RPE, the discrepancy between gene expression and the secreted IL-6 suggests a potential failure in either protein translation, processing, or trafficking to the plasma membrane.

The long-term goal of this study was to validate iPSC-RPE derived from AMD patients as a “disease in a dish” in vitro platform for studying AMD disease mechanism and testing drugs. In our previous work, we showed that primary RPE cultures from donors with AMD display reduced mitochondrial function [16] and that these defects could be partially rescued with drugs that either protected or increased healthy mitochondria [30,36]. A reduction in mitochondrial function that was attributed to defects in autophagy has also been reported in primary RPE cultures from AMD donors [37].

While studies in primary RPE cell cultures have revealed important details about AMD disease mechanism, the limited availability of adult RPE from donors graded for the stage of AMD and the finite cell number from each donor prohibits their global use. iPSC-RPE provides a viable alternative with a number of advantages including a nearly unlimited number of RPE cells, the availability of patient-specific cell lines, and the potential for personalized medicine, as iPSC-RPE can be generated from a variety of somatic cells (i.e., blood cells, skin, corneal epithelium) obtainable from living patients. However, caveats have been raised regarding the suitability of using iPSC-RPE to study AMD, including how closely iPSC-RPE may reflect native RPE and the potential importance of the somatic cells used for reprogramming. Results from published studies have found similarities in phenotypic and functional characteristics when comparing iPSC-RPE derived from adult RPE or skin fibroblasts [37] and comparing iPSC-RPE derived from fetal (or adult) RPE or cornea [38]. There are also questions about how closely iPSC-RPE can replicate AMD since this disease develops mainly in older individuals over many years and involves an accumulation of environmental insults in the retina. In contrast, iPSC-RPE have not been exposed to the same diseased environment as RPE in situ. However, the iPSC-RPE do share the genotype of the donors and recent publications (summarized below), as well as the results from this study provide increasing confidence that iPSC-RPE can provide a valid model system for studying AMD, especially when investigating the impact of the donors’ genetic background.

Several studies have provided mechanistic insight into AMD disease pathology. Consistent with our findings, two groups have reported reduced mitochondrial function in iPSC-RPE from AMD patients [21,23]. Notably, a dramatic AMD-associated phenotype was observed when cells were grown on nitrated membrane, a condition that mimics the aged Bruch’s membrane [23]. This result supports the idea that stress can be used to reveal important phenotypes, as we observed when we stressed the mitochondria in this study (Figure 2A). Other studies using iPSC-RPE have focused on specific risk SNPs associated with AMD to investigate how the presence of risk alleles lead to pathology. Studies of the *ARMS2/HTRA1* (rs1040924/rs11200638) genes, which confers the highest risk for AMD, revealed reduced antioxidant defense and greater susceptibility to oxidative damage [24]. A second study showed iPSC-RPE from AMD patients exhibited higher expression of complement and inflammatory factors and that the presence of the ARMS2/HTRA1 risk allele magnified these results [25]. One other group, focused on the CFH high risk SNP, has reported increased inflammation and cellular stress, reduced autophagy, and deposition of lipid droplets and “drusen-like” deposits in iPSC-RPE from diseased donors harboring the high-risk allele [26]. A subsequent study associated the CFH high-risk allele with damaged lysosomes due to accelerated complement activity [27]. Taken together, these studies support the idea that iPSC-RPE provide a valid model system to investigate the effect of a specific genetic background associated with increased risk of AMD.

It is important to mention several key differences in experimental design and methods that varied between our work and others using iPSC-RPE to study AMD that could influence study results and conclusions. Our study used eight or more donors per group, while previous studies were limited to four or fewer donors per group [21,23,25,26,27,39]. A robust sample size helps to validate conclusions, especially when there is high variability among the sample population. Another strength of our study was our use of the Minnesota Grading System [28] as a way to determine the stage of AMD, which provided us an opportunity to investigate early disease. Other studies have used cells from patients already in the late stages of AMD [23,25,26,27,37,39]. Growth conditions of the iPSC-RPE also vary from study to study. For example, cells grown on different types of matrix can respond differently. The use of nitrite-modified matrix [23], human placental extracellular matrix [25], or hESC-qualified Matrigel^®^ matrix (in this study) create their own microenvironment and cellular response, which can make it challenging to compare results between experimental studies.

We found that iPSC-RPE harboring the CFH high-risk allele had significantly reduced mitochondrial function (Figure 2), indicating a link between CFH and mitochondrial regulation. Two recent studies support this idea. A significant decline in retinal ATP and a significant increase in heat shock protein 60 (Hsp60), a marker of mitochondrial stress, was observed in CFH knockout (Cfh−/−) mice compared to WT mice [40]. In hTERT-RPE1 cells, silencing CFH expression using siRNA resulted in impaired mitochondrial respiration and upregulation of the mitophagy (mitochondria-specific autophagy) genes, PTEN-induced kinase-1 (PINK1) and PARKIN, compared to the negative control [41]. Additional studies show CFH also influences other cell processes. Young Cfh−/− mice have significantly disrupted and delayed retinal development [42] and aged Cfh−/− mice have decreased visual acuity compared to age-matched control mice [43]. Similar to Cfh−/− mice, transgenic mice expressing human H402 CFH fed a high fat diet also had visual dysfunction [44]. These results suggest a role for CFH that extends beyond regulation of the extracellular complement cascade and inflammation.

**Figure 6:**
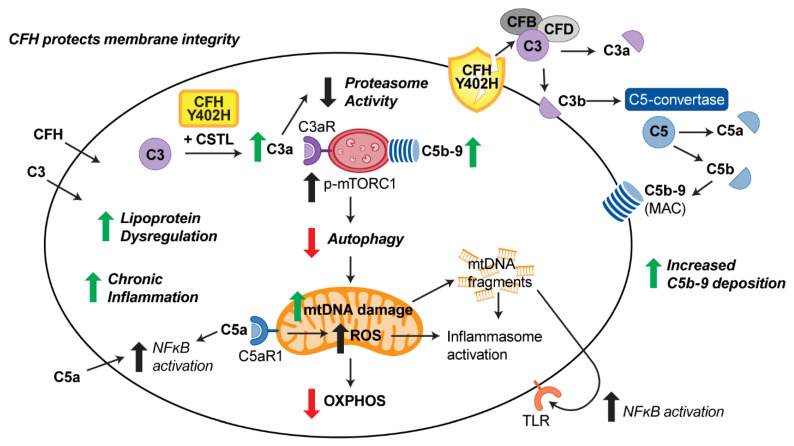
Model of CFH regulation of RPE function and putative genotype-specific effects. Extracellular CFH protects cell membranes and inhibits C3 conversion and subsequent deposition of the MAC complex. Intracellular complement can regulate pathways involved in degradation (proteasome and lysosome/autophagy) and mitochondrial homeostasis. (Green and red arrows indicate Y402H-dependent changes). In Y402H iPSC-RPE, enhanced C3a turnover, increased C5b9 internalization with lysosomal deposition, and reduced autophagy disrupts degradation of protein aggregates and damaged organelles. Reduced oxidative phosphorylation and increased mtDNA damage indicates a disruption in mitochondrial homeostasis. Accumulation of lipoprotein and increased inflammation further exacerbates RPE dysfunction.

The most widely accepted mechanism by which the CFH Y402H variant increases the risk for AMD involves its reduced ability to inhibit extracellular C3 activation, resulting in chronic inflammation and damage to eye tissue, including RPE and choroid [6]. While the mechanism for CFH regulation of mitochondrial function in RPE cells is unclear, it may involve a newly emerging role for an intracellular active complement system that regulates multiple processes, such as metabolism, inflammation, and cell survival [45]. Even though the liver is the main source of systemic complement proteins, the locally produced ocular complement proteins provide the greatest contribution to AMD risk [46]. The two sources of intracellular complement proteins include their localized expression in the RPE ((Figure 5), [6,47]) and the internalization of complement proteins from the extracellular milieu [48,49,50].

New information about intracellular complement that is emerging from multiple studies in RPE and other cell types provides insight into the putative mechanism linking mitochondrial function and CFH (Figure 6). As discussed previously, extracellular CFH inhibits C3 conversion to its active products, thereby preventing deposition of the MAC complex on the cell’s outer membrane. More recent studies have also shown that extracellular CFH also protects RPE membrane integrity [41,51]. Intracellular CFH acts as a cofactor for cathepsin L (CTSL) cleavage of C3 into C3a and C3b [49,52]. Multiple intracellular roles for C3a have been described, including stimulation of intracellular C3aR signaling in the lysosomes [53], leading to sustained mechanistic target of rapamycin (mTOR) activity [53,54] and reduced autophagy. Interaction of C3a with the proteasome, which is the cells other major degradation pathway, reduces its activity [50]. Thus, C3a can regulate overall cellular function by disrupting pathways involved in cell signaling and clearance of damaged proteins and organelles. The reduced ability of the CFH Y402H variant to inhibit C3 activation enhances the risk of this disruption. Mitochondrial function could also be affected by the production of reactive oxygen species (ROS) resulting from the activation of the mitochondrial-localized C5aR1 by C5a [55]. C5a can also activate NFκB and upregulate inflammation [56]. Taken together, these results show how the intracellular complement system engages in crosstalk with multiple cell pathways that regulate cell homeostasis [57].

In iPSC-RPE harboring the high-risk CFH genotype, both increased extracellular deposition on the outer membrane [51] as well as increased internalization and deposition of the C5b-9 MAC complex on the lysosomes [27] has been reported. Lysosomes in CFH Y402H iPSC-RPE also appeared swollen and had lower cathepsin D activity, changes that could contribute to the observed reduction in autophagy flux [27]. Additionally reported was an accelerated turnover of C3 to C3a [27], which could be the relevant upstream change initiating the defects in the lysosome-autophagy pathway. Decreased autophagy could allow for accumulation of damaged mitochondria and may be responsible for the observed reduction in mitochondrial function (Figure 2) and increased mtDNA damage previously reported in RPE from donors harboring the CFH Y402H risk allele [17]. Release of damaged mtDNA can cause inflammation via either intracellular NLRP3 inflammasome activation [58,59], or binding of secreted mtDNA to toll-like receptors (TLR) that activate NFκB signaling [60]. Lipid droplet accumulation, increased inflammation and cell stress were also linked to the CFH Y402H genotype in iPSC-RPE [26]. Although the functional consequences of how the CFH high-risk variant affects intracellular complement activity is still incomplete, these new reports provide insight into its effect on multiple cell pathways that could impact cell homeostasis.

## 5. Conclusions

Development of a comprehensive in vitro model for studying AMD remains a challenge due to the multifactorial nature of the disease, with both environment and genetics contributing to an individual’s risk. However, results from multiple studies suggest that iPSC-RPE are a valid model system for studying AMD as the phenotypes revealed reflect both the donor’s disease state and their disease risk genotype. Additionally, application of this model system as a platform for patient-specific drug testing and development will be an invaluable asset in finding new approaches to treat AMD. As an example, results from the current study suggest a patient harboring the CFH Y402H genotype may benefit from therapies aimed at improving retinal mitochondrial function potentially by targeting the intracellular complement pathway. This idea is particularly relevant considering the number of therapeutics currently in clinical trials for AMD that disrupt the complement cascade by either inhibiting complement components (i.e., C5, C3) or increasing molecules that inhibit the complement cascade (CFH, CD59) [61].

## Figures and Tables

**Table 1:** Description of iPSC-RPE lines and donor characteristics.

iPSC-RPE Line ID	Line #	Age ^a^/Gender ^b^	MGS Stage ^c^	CFH Genotype ^d^	Figures Using Data from Specific Lines
MGS1-1473-1D3	1	71/M	MGS1	TT	1B–D, 2B–F, 4C–E, 5C–E
MGS1-1473-2B6	2	71/M	MGS1	TT	1A–D, 2A–F, 3A–D, 4A–E, 5A–E
MGS1-0237-2C3	3	77/F	MGS1	TT	1B–D, 2B–F, 4A–E, 5A–D
MGS1-0237-3A3	4	77/F	MGS1	TT	1B–D, 2B–F, 4A–B, 5A–D
MGS1-0698-1A2	5	73/F	MGS1	TT	2A, 3A–D, 4A–B, 5A–B
MGS1-0698-1A3	6	73/F	MGS1	TT	4A–B, 5A–B
MGS1-1418-1A2	7	54/M	MGS1	TT	1B–D, 2B–F, 4C–E, 5C–E
MGS1-1580-1A4	8	57/M	MGS1	TT	1B–D, 2B–F, 3A–D, 4C–E, 5C–E
MGS1-1686-C	9	73/M	MGS1	TT	5A–B,E
MGS1-1345-1C4	10	66/M	MGS1	CT	1B–D, 2B–F, 3A–D, 4C–E, 5C–D
MGS1-0027-1A3	11	80/M	MGS1	CT	1B–D, 2A–F, 3A–D, 4A–E, 5A–E
MGS1-0027-1B3	12	80/M	MGS1	CT	1B–D, 2B–F, 3A–D, 4A–E, 5A–E
MGS1-0553-2	13	68/M	MGS1	CT	1A–D, 2A–F, 3A–D, 4A–E, 5A–E
MGS1-1230-C	14	84/F	MGS1	CT	5A–B,E
MGS2-1747-1A1	15	76/M	MGS2	CT	1B–D, 4A–E, 5A–E
MGS2-1747-2A4	16	76/M	MGS2	CT	1B–D, 2B–F, 4A–E, 5A–E
MGS2-1759-1E3	17	63/F	MGS2	TT	2B–F, 4A–E, 5A–B
MGS2-1759-1E6	18	63/F	MGS2	TT	2B–F, 4A–E, 5A–B
MGS2-1826-1C2	19	80/M	MGS2	CT	1B–D, 2B–F, 3A–D, 4A–B, 5A–E
MGS2-2360-3	20	58/M	MGS2	CT	1B–D, 2A–F, 3A–D, 4A–E, 5A–E
MGS2-0024-2	21	75/F	MGS2	CC	1B–D, 2B–F, 3A–D, 4A–E, 5A–E
MGS2-0024-3	22	75/F	MGS2	CC	1B–D, 2B–F, 3A–D, 4A–E, 5A–E
MGS2-0590-1	23	66/M	MGS2	CT	1A–D, 2B–F, 3A–D, 4A–E, 5A–E
MGS2-0590-2	24	66/M	MGS2	CT	2A–F, 3A–D, 4A–E, 5A–B
MGS2-1935-C	25	80/F	MGS2	TT	5A–B,E
MGS2-1825-3	26	70/F	MGS2	CC	5A–B,E
MGS3-1775-6B4	27	85/F	MGS3	CC	1B–D, 2B–F, 4C–E, 5C–D
MGS3-0878-1B1	28	79/M	MGS3	CT	1B–D, 2B–F, 4A–E, 5A–D
MGS3-0878-1C4	29	79/M	MGS3	CT	1B–D, 2B–F, 3A–D, 4A–E, 5A–D
MGS3-1424-1A4	30	72/F	MGS3	CT	1B–D, 2A–F, 3A–D, 4A–E, 5A–D
MGS3-1424-1A5	31	72/F	MGS3	CT	1B–D, 2B–F, 4C–E, 5C–D
MGS3-2020-1A6	32	84/F	MGS3	TT	1B–D, 2A–F, 3A–D, 4A–E, 5A–E
MGS3-0277-1C1	33	80/F	MGS3	TT	1B–D, 2B–F, 3A–D, 4A–E, 5A–E
MGS3-0277-1C5	34	80/F	MGS3	TT	1B–D, 2B–F, 4A–E, 5A–E
MGS3-0824-2	35	85/M	MGS3	TT	1A, 2A–F, 3A–D, 4A–E, 5A–E
MGS3-0011-1	36	83/F	MGS3	TT	2B–F, 3A–D, 4A–E, 5A–B
MGS3-0011-3	37	83/F	MGS3	TT	1B–D, 2A–F, 3A–D, 4A–E, 5A–E
MGS3-0276-1	38	75/F	MGS3	CC	1B–D, 2B–F, 3A–D, 4A–E, 5A–E

^a^ Age of donor, in years, from whose conjunctiva cells were used to generate iPSC-RPE. ^b^ Gender of donor. F = female, M = male. ^c^ Minnesota Grading System (MGS) was used to evaluate the stage of AMD in eye bank eyes [28]. No AMD = MGS1; AMD = MGS2 (early AMD) and MGS3 (intermediate AMD). ^d^ Complement Factor H (CFH) genotype for rs106117; low risk = TT, high risk = CT and CC.

## Data Availability

Data used to support the findings in this study are contained within this article and Supplementary Material.

## Supplementary Materials

The following are available online at https://www.mdpi.com/article/10.3390/cells10040789/s1, Figure S1: Determining Cellular Metabolic Function using XFe96 Extracellular Flux Analyzer, Figure S2: Comparing mitochondrial function in iPSC-RPE, Figure S3: Characterization of iPSC-RPE, Figure S4: Concordance between iPSC-RPE lines generated the same donor, Table S1: List of primer sequences for Real Time PCR analysis.
