# Supplementary material for: Impaired Mitochondrial Function in iPSC-Retinal Pigment Epithelium with the Complement Factor H Polymorphism for Age-Related Macular Degeneration
Source: Cells. 2021 Apr 2;10(4):789. doi: 10.3390/cells10040789 (PMC8066149; doi:10.3390/cells10040789)
Supplement: Supplementary file 1 [file cells-10-00789-s001.pdf]

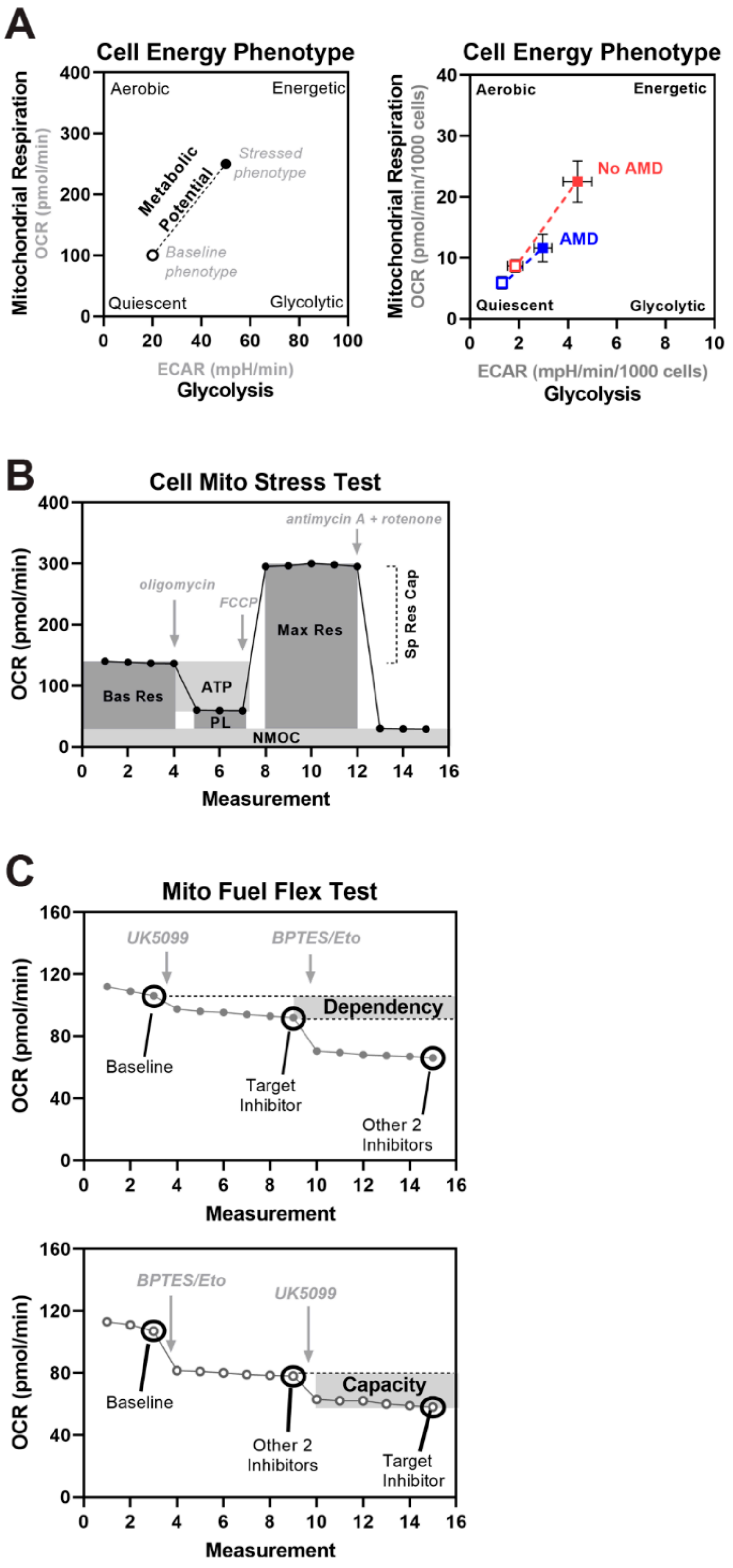
A
Cell Energy Phenotype
Mitochondrial Respiration
OCR (pmol/min)
Aerobic
Energetic
Metabolic Potential
Stressed phenotype
Baseline phenotype
Quiescent
Glycolytic
ECAR (mpH/min)
Glycolysis
Cell Energy Phenotype
Mitochondrial Respiration
OCR (pmol/min/1000 cells)
No AMD
AMD
ECAR (mpH/min/1000 cells)
B
Cell Mito Stress Test
OCR (pmol/min)
oligomycin
FCCP
antimycin A + rotenone
Bas Res
ATP
PL
Max Res
Sp Res Cap
NMOC
Measurement
C
Mito Fuel Flex Test
OCR (pmol/min)
UK5099
BPTES/Eto
Dependency
Baseline
Target Inhibitor
Other 2 Inhibitors
Measurement
BPTES/Eto
UK5099
Capacity
Baseline
Other 2 Inhibitors
Target Inhibitor
Measurement

Figure S1. Determining Cellular Metabolic Function using XFe96 Extracellular Flux Analyzer. Graphs show trace and methods for calculating metabolic parameters. Arrows indicate time of injection for specific reagents. (A) Data associated with XF Cell Energy Phenotype Test. (B) Trace associated with Cell Mito Stress Test. Analysis of oxygen consumption rate (OCR) following injections of oligomycin, FCCP, and antimycin A + rotenone to perturb mitochondrial function. Calculation of the basal respiration (Bas Res), maximal respiration (Max Res), spare respiratory capacity (Sp Res Cap), ATP-linked respiration (ATP), Proton Leak (PL), and Non-Mito Oxygen Consumption (NMOC) are shown. (C) Example traces associated with Mito Fuel Flex Test. Analysis of OCR following injections of inhibitors targeted to mitochondrial fuel pathways.

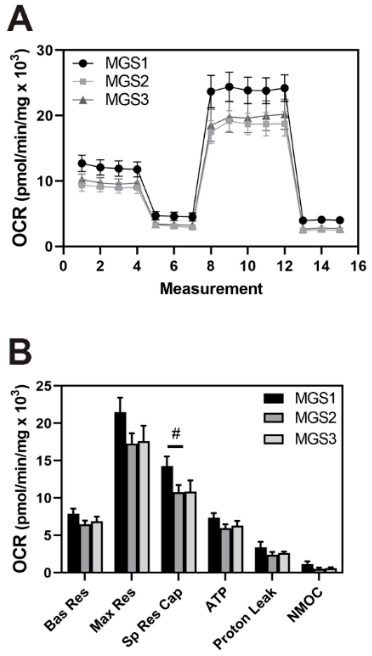

Figure S2. Comparing mitochondrial function in iPSC-RPE. (A) Trace from the CMST from multiple donors. (B) Parameters of mitochondrial function calculated from data in A. #p<0.01 determined by One Way ANOVA with Tukey's multiple comparison. MGS1 (No AMD) 8 donors, 11 lines; MGS2 (early AMD) 6 donors, 9 lines; MGS3 (intermediate AMD) 8 donors, 12 lines). Bar graphs are mean ± SEM.

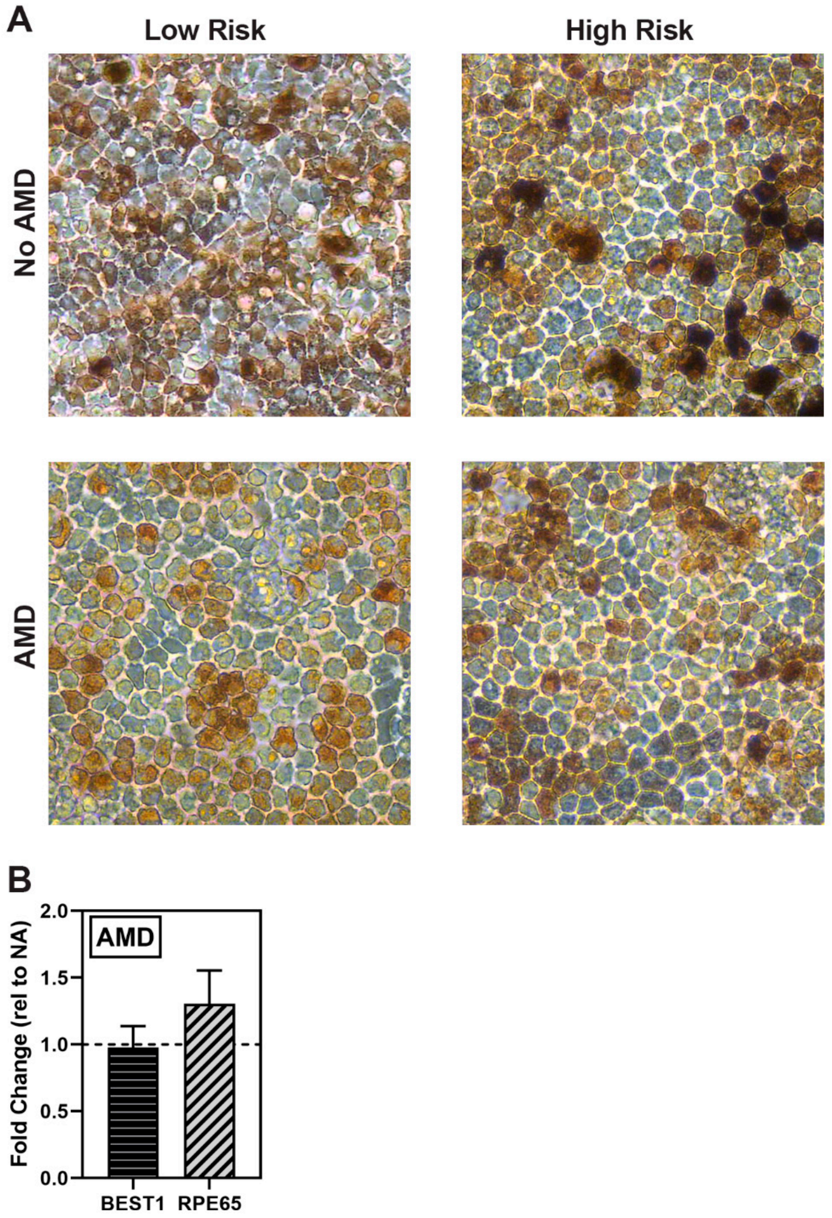

Figure S3. Characterization of iPSC-RPE. (A) Phase contrast microscopy images shows confluent iPSC-RPE derived from No AMD and AMD donors form a monolayer with cobblestone appearance and pigmentation. (B) RT-qPCR analysis of prototypic RPE genes was performed on iPSC-RPE derived from No AMD (4 donors, 7 lines) and AMD (12 donors, 17 lines) donors. Data shown are relative to No AMD (dashed line). Bar graphs are mean ± SEM.

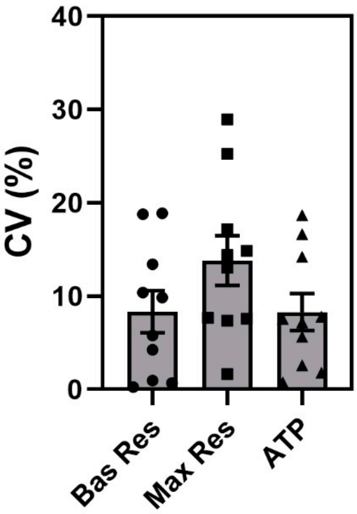

Figure S4. Determining the degree of concordance between iPSC-RPE lines generated from the same donor. To compare two iPSC-RPE lines from the same donor (n=10 total donors; 3 No AMD, 7 AMD), coefficient of variance (CV = (stdev/mean)*100) was calculated from the results of mitochondrial parameters measured in the CMST assay. Bar graphs are mean ± SEM.

Table S1. List of primer sequences for Real Time PCR analysis

Gene	Forward Primer	Reverse Primer
BEST1	TAGAACCATCAGCGCCGTC	TGAGTGTAGTGTGTATGTTGG
RPE65	CGTATGGACTTGGCTTGAATC	CTGGGTGAGAAACAAAGATGG
GAPDH	GAGTCAACGGATTTGGTCGT	GACAAGCTTCCCGTTCTCAG
C3	GCTACATCATCGGGAAGGAC	CTGGCATTGTTTCTGGTTCTC
C3AR	CCTGCTGATGTGGTCTCACCT	CCTTGTGGTAGCTCAGACTCGT
C5AR1	GCCCAGGAGACCAGAACAT	TATCCACAGGGGTGTTGAGG
CFB	CCCTATGCTGACCCCAATAC	GATTACACCAACTTGAATGAAACG
CFHv1	AACAGATTGTCTCAGTTTACCTAGC	ACCCGCCTTATACACATCCTTC
CFHv2	CTTTACCCTCTGAACTTCTGATCG	TCTGGCTGGAATAATACACACATAAC
CFI	TTGGATTCTGACTGCTGCAC	TTGTCCATATTTGGTAACGATGA
CD46	TGGCTACCTGTCTCAGATGACG	GCATCTGATAACCAAACTCGTAAG
CD55	CTGCTGGTGCTGTTGTGC	TCCTCGGGAAAACTTGTACG
CD59	GAGCCCAGGGAGGGAAAGGTT	CGAGGTTAAGGCAAAACCCTACGG
TNFA	CTCTTCTGCCTGCTGCACTTTG	ATGGGCTACAGGCTTGTCACTC
MCP1	CTCATAGCAGCCACCTTCATTC	TCACAGCTTCTTTGGGACACTT
IL1B	CCACAGACCTTCCAGGAGAA	GTGATCGTACAGGTGCATCG
IL6	GGTACATCCTCGACGGCATCT	GTGCCTCTTTGCTGCTTTCAC
MT-ND2	AACCCTCGTTCCACAGAAGCT	GGATTATGGATGCGGTTGCT
MT-ND4	CCCACTCCCTCTTAGCCAATATT	TAGGCCCACCGCTGCTT
MT-ND6	GCCCCCGCACCAATAGGATCCTCCC	CCTGAGGCATGGGGGTCAGGGGT
MT-CYB	CCCACCCTCACACGATTCTTA	TTGCTAGGGCTGCAATAATGAA
MT-CO2	ACCAGGCGACCTGCGACTCCT	ACCCCCGGTCGTGTAGCGGT
MT-ATP6	TTATGAGCGGGCACAGTGATT	GAAGTGGGCTAGGGCATTTTT
HPRT1	TGCAGACTTTGCTTTCCTTGGTCAGG	CCAACACTTCGTGGGGTCCTTTTCA
ARBP	CGACCTGGAAGTCCAACTAC	ATCTGCTGCATCTGCTTG
